# Hypoxia-inducible factors in breast cancer: prognostic indicators and emerging biomarkers: narrative review

**DOI:** 10.1097/MS9.0000000000003500

**Published:** 2025-06-16

**Authors:** Emmanuel Ifeanyi Obeagu

**Affiliations:** Department of Biomedical and Laboratory Science, Africa University, Mutare, Zimbabwe

**Keywords:** angiogenesis, biomarkers, breast cancer, hypoxia-inducible factor (HIF), therapeutic targeting

## Abstract

The hypoxia-inducible factor (HIF) pathway is a critical regulator of cellular responses to low oxygen conditions, which are prevalent in solid tumors like breast cancer. Under hypoxic conditions, HIF transcription factors, particularly HIF-1α and HIF-2α, orchestrate various tumor-promoting processes, including angiogenesis, metabolic reprogramming, and metastasis. These adaptive responses contribute significantly to tumor progression and resistance to conventional therapies. As such, understanding the molecular mechanisms underlying the HIF pathway offers valuable insights into breast cancer biology and provides a foundation for the development of novel therapeutic strategies. Recent advancements in the identification of biomarkers associated with the HIF pathway have shown potential for improving prognosis and guiding therapeutic decisions. Biomarkers such as HIF-1α, vascular endothelial growth factor, glucose transporter 1, and carbonic anhydrase IX are linked to hypoxia-driven tumor behaviors and may serve as indicators of disease aggressiveness and patient outcomes. Their integration into clinical practice could enable more precise stratification of patients for HIF-targeted interventions, facilitating the move toward personalized treatment regimens in breast cancer care.

## Introduction

Breast cancer remains one of the most common and deadly forms of cancer worldwide. Despite significant advancements in early detection, treatment modalities, and supportive care, the clinical outcomes for many breast cancer patients are still unsatisfactory, particularly for those diagnosed with advanced or metastatic disease. A major contributing factor to the poor prognosis of breast cancer is the dynamic tumor microenvironment, which includes various stressors such as hypoxia. Hypoxia, or low oxygen availability, is a hallmark of solid tumors due to the imbalance between the rapid growth of tumor cells and the insufficient development of blood vessels (angiogenesis). Hypoxia influences the behavior of tumor cells and is associated with aggressive tumor phenotypes, including metastasis, therapy resistance, and poor survival outcomes^[1,2]^. The hypoxia-inducible factor (HIF) pathway is a central regulator of cellular responses to hypoxic conditions. The HIF family consists of oxygen-sensitive transcription factors, with HIF-1α and HIF-2α being the most studied in cancer. Under normal oxygen conditions, HIF-α subunits are rapidly degraded through the action of prolyl hydroxylase domain (PHD) containing enzymes, which mark them for proteasomal degradation. However, during hypoxia, the activity of PHDs is reduced, allowing HIF-α subunits to stabilize, dimerize with HIF-β, and translocate to the nucleus where they initiate the transcription of genes involved in adaptive responses to low oxygen levels. These include genes that regulate angiogenesis, glucose metabolism, cell survival, and invasion, all of which play a critical role in tumor growth and metastasis^[3,4]^.

In breast cancer, the activation of the HIF pathway is crucial for tumor progression. Hypoxia-induced HIF signaling promotes several oncogenic processes, including angiogenesis (formation of new blood vessels), altered metabolism, and epithelial-to-mesenchymal transition (EMT), all of which contribute to tumor growth and spread. Angiogenesis, driven by the upregulation of vascular endothelial growth factor (VEGF), is essential for providing oxygen and nutrients to growing tumors. Additionally, HIF signaling reprograms tumor metabolism toward glycolysis, a phenomenon known as the Warburg effect, which allows cancer cells to survive and proliferate in the low-oxygen environment of tumors. The involvement of HIF in these processes makes it a critical player in breast cancer pathophysiology^[5,6]^. Moreover, HIF pathway activation also plays a significant role in regulating the tumor microenvironment, influencing immune responses and resistance to therapy. Hypoxic regions within tumors can lead to immune evasion by suppressing the function of immune cells and creating a pro-tumorigenic environment. Furthermore, the HIF pathway has been implicated in the development of resistance to chemotherapy and radiotherapy. Tumors with high levels of HIF activity are often more resistant to these treatments, as HIF-induced changes in metabolism and cell survival mechanisms allow tumor cells to withstand the damaging effects of therapy^[7,8]^. Given its central role in breast cancer progression, the HIF pathway has emerged as a promising target for therapeutic intervention. Researchers have focused on identifying small molecule inhibitors and other therapeutic agents that can block the activity of HIF-α or prevent its stabilization. Additionally, therapies aimed at inhibiting downstream targets of HIF, such as VEGF, have shown promise in preclinical and clinical studies. However, the clinical application of HIF-targeted therapies remains challenging, as the complexity of HIF signaling, its redundancy with other pathways, and the potential for compensatory mechanisms make it difficult to achieve sustained therapeutic effects^[9,10]^. In recent years, the identification and validation of biomarkers associated with the HIF pathway have become a key area of research. These biomarkers, including HIF-1α, VEGF, carbonic anhydrase IX (CAIX), and glucose transporter 1 (GLUT-1), have the potential to serve as diagnostic tools, prognostic indicators, and therapeutic targets in breast cancer. For example, the overexpression of HIF-1α has been associated with aggressive tumor behavior, poor prognosis, and resistance to therapy in breast cancer patients. Additionally, the detection of HIF-related biomarkers in tissue samples or in circulating tumor cells (CTCs) could aid in early detection, monitoring of disease progression, and assessment of treatment efficacy^[11,12]^.

## Aim

The aim of this review is to explore the role of the HIF pathway in breast cancer progression, focusing on its molecular mechanisms, associated biomarkers, and potential therapeutic opportunities.

## Review methods

This review is a narrative synthesis of the current evidence on HIFs as diagnostic and prognostic biomarkers in breast cancer. A comprehensive literature search was conducted across PubMed, Scopus, and Web of Science databases, covering studies published between January 2000 and March 2025. Search terms included combinations of “hypoxia-inducible factors,” “HIF,” “breast cancer,” “biomarkers,” “diagnosis,” “prognosis,” and “HIF inhibitors.” Only English-language articles were considered, including original research, clinical studies, and relevant reviews. To ensure the novelty and relevance of this work, we critically compared our review to existing literature, including the comprehensive review by Luo *et al*[7] While Luo *et al*[7] provided an in-depth exploration of HIF inhibitors and therapeutic implications, our review uniquely contributes by presenting a biomarker-centered perspective with a clinical orientation. Specifically, we map HIF-related markers across different breast cancer subtypes, outline their diagnostic and prognostic implications, and highlight their potential translational utility. This structured approach offers clinicians and researchers a more focused understanding of the biomarker landscape associated with HIF signaling in breast cancer.

## The HIF pathway: mechanisms in breast cancer

The HIF pathway is a key regulator of cellular adaptation to low oxygen levels (hypoxia), which commonly occur in the tumor microenvironment. Hypoxia is a hallmark of many solid tumors, including breast cancer, due to the rapid growth of tumor cells outpacing the development of blood vessels. Under normoxic conditions, HIF-α subunits (HIF-1α and HIF-2α) are continuously degraded through the action of PHD-containing enzymes that hydroxylate specific proline residues on HIF-α. This hydroxylation facilitates recognition by the von Hippel-Lindau tumor suppressor protein, marking the HIF-α subunits for proteasomal degradation. However, during hypoxia, PHDs become inactive due to low oxygen availability, leading to the stabilization of HIF-α subunits. These stabilized HIF-α subunits then dimerize with HIF-β and translocate to the nucleus, where they bind to hypoxia-responsive elements (HREs) in the promoter regions of target genes, initiating transcriptional activation of genes involved in the adaptive response to hypoxia^[13,14]^. In breast cancer, HIF-1α and HIF-2α play distinct but complementary roles in regulating tumor progression. HIF-1α is primarily associated with the early stages of tumor development and is more widely studied for its role in initiating adaptive responses to hypoxia. It activates genes involved in angiogenesis, such as VEGF, which promotes the formation of new blood vessels to supply the growing tumor with oxygen and nutrients. Additionally, HIF-1α induces the expression of genes involved in glucose metabolism, such as GLUT-1 and glycolytic enzymes, promoting the Warburg effect – an altered metabolic pathway that enables cancer cells to thrive in hypoxic conditions. By enhancing glycolysis, HIF-1α facilitates the metabolic reprogramming necessary for tumor cell survival and growth in the oxygen-deprived tumor microenvironment^[15,16]^.

**HIGHLIGHTS**

- Hypoxia-inducible factor (HIF) and tumor microenvironment: HIFs reshape the tumor microenvironment, promoting immune evasion and cancer progression.
- HIF in early detection: Circulating HIF markers may serve as noninvasive tools for early breast cancer diagnosis.
- HIF-driven metabolic reprogramming: HIF signaling alters cellular metabolism, supporting tumor survival under low oxygen conditions.
- HIF and hormone receptor status: HIF expression varies across breast cancer subtypes, influencing treatment response and prognosis.
- Combination therapies targeting HIF: Integrating HIF inhibitors with chemotherapy or immunotherapy may enhance treatment efficacy in breast cancer.

**Figure 1.**
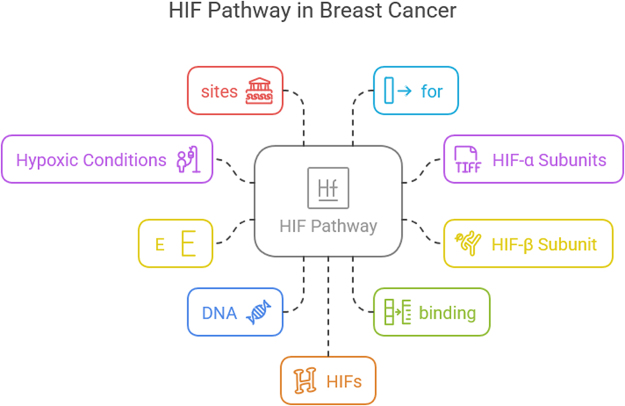
The HIF pathway in breast cancer.

HIF-2α, while less studied than HIF-1α, also contributes to tumor progression and is often associated with more aggressive breast cancer subtypes. HIF-2α has been implicated in promoting tumor cell invasion and metastasis by regulating the expression of genes involved in EMT, a process that allows tumor cells to acquire migratory and invasive properties. EMT is a critical step in the metastatic spread of cancer, and HIF-2α has been shown to upregulate genes like Snail and Twist, which are key transcriptional regulators of EMT. Moreover, HIF-2α also enhances the expression of matrix metalloproteinases (MMPs), enzymes that degrade the extracellular matrix, facilitating tumor cell invasion into surrounding tissues and ultimately leading to metastasis. The distinct roles of HIF-1α and HIF-2α in regulating different aspects of tumor biology make them both essential players in breast cancer progression[17]. In addition to regulating angiogenesis, metabolism, and invasion, the HIF pathway also influences immune responses within the tumor microenvironment. Hypoxia can create an immunosuppressive environment by altering the function of immune cells, such as T cells and macrophages, and by inducing the expression of immune checkpoint molecules like PD-L1. This allows the tumor to evade immune surveillance, further promoting tumor growth and resistance to therapy. HIF-1α, in particular, has been shown to promote the expression of immune-related genes, including those involved in immune cell recruitment and the suppression of anti-tumor immune responses. The ability of the HIF pathway to modulate the immune landscape of tumors suggests that targeting this pathway may enhance the effectiveness of immunotherapies and help overcome immune evasion mechanisms in breast cancer[18]. Furthermore, the HIF pathway contributes to therapy resistance in breast cancer. Tumors with high levels of HIF activation are often more resistant to chemotherapy and radiotherapy. This is partly due to the metabolic adaptations that HIF induces, which allow tumor cells to survive under harsh conditions. For instance, HIF-1α-mediated upregulation of glycolysis can enable cancer cells to survive in low-oxygen environments that would otherwise lead to cell death. Moreover, HIF signaling promotes the expression of genes involved in cell survival, such as BCL-2, and anti-apoptotic factors, which help tumor cells evade programmed cell death. These adaptations render tumors more resilient to conventional therapies, highlighting the need for HIF-targeted treatments to overcome therapy resistance in breast cancer (Fig. 1)[19].

## Biomarkers of the HIF pathway in breast cancer

The identification and validation of biomarkers associated with the HIF pathway in breast cancer have become a pivotal area of research. These biomarkers provide critical insights into the molecular mechanisms driving tumor progression, help stratify patients according to risk, and can potentially guide therapeutic interventions. HIF pathway biomarkers are typically involved in key processes such as angiogenesis, metabolism, cell survival, invasion, and immune evasion, all of which are crucial for the malignant phenotype of breast cancer (Table 1)[20].

1. HIF-1α and HIF-2α: HIF-1α and HIF-2α are the principal transcription factors activated in response to hypoxia and are among the most direct biomarkers of the HIF pathway. Both isoforms of HIF have been implicated in various aspects of breast cancer biology, including tumor progression, metastasis, and therapy resistance. Overexpression of HIF-1α is often associated with aggressive breast cancer phenotypes and poor prognosis. High levels of HIF-1α have been linked to increased tumor size, higher grade, and a greater likelihood of metastasis. Similarly, elevated HIF-2α expression is often found in more aggressive subtypes of breast cancer, such as triple-negative breast cancer (TNBC), and has been shown to promote tumor invasion and metastasis. The detection of HIF-1α and HIF-2α in tumor biopsies or CTCs may serve as indicators of tumor aggressiveness and metastasis risk[21].
2. VEGF: VEGF is a well-known downstream target of HIF-1α and plays a crucial role in promoting angiogenesis, the process of forming new blood vessels to supply the growing tumor with oxygen and nutrients. The expression of VEGF is strongly upregulated under hypoxic conditions through HIF-1α-mediated transcriptional activation. In breast cancer, high VEGF levels are commonly associated with poor prognosis, increased tumor vasculature, and enhanced metastatic potential. As VEGF plays a significant role in angiogenesis, it is frequently used as a biomarker for assessing tumor vascularity and predicting response to anti-angiogenic therapies. VEGF-targeted therapies, such as bevacizumab, have been investigated in clinical trials, and its expression remains a potential biomarker for patient selection in these treatments[22].
3. GLUT-1: GLUT-1 is another important gene regulated by HIF-1α in response to hypoxia. It is responsible for the increased glucose uptake that supports the Warburg effect, a metabolic shift observed in many tumors where cells preferentially utilize glycolysis over oxidative phosphorylation for energy production, even in the presence of oxygen. In breast cancer, GLUT-1 overexpression has been associated with increased glycolytic activity and tumor progression. High GLUT-1 expression is commonly found in aggressive breast cancer subtypes, such as basal-like and TNBC, and correlates with poor prognosis. GLUT-1 has been proposed as a potential biomarker for assessing metabolic activity and identifying patients who might benefit from therapies targeting the metabolic vulnerabilities of tumors[23].
4. CAIX: CAIX is another prominent biomarker of the HIF pathway. It is a transmembrane protein that is upregulated by HIF-1α under hypoxic conditions. CAIX plays a role in maintaining pH homeostasis within tumor cells by catalyzing the conversion of carbon dioxide to bicarbonate, which helps neutralize the acidic microenvironment created by excessive lactic acid production during glycolysis. CAIX is widely considered a marker of tumor hypoxia and is frequently overexpressed in breast cancer, particularly in more aggressive tumors. Elevated CAIX expression has been correlated with poor clinical outcomes, making it a promising candidate for monitoring tumor progression, identifying hypoxic tumor regions, and predicting the response to therapies aimed at reducing tumor acidity or targeting CAIX directly[24].
5. MMPs: MMPs, particularly MMP-2 and MMP-9, are key regulators of the extracellular matrix (ECM) remodeling process, which is crucial for tumor cell invasion and metastasis. HIF-1α has been shown to upregulate the expression of various MMPs, facilitating the breakdown of the ECM and enabling tumor cells to invade surrounding tissues. In breast cancer, high MMP activity is often associated with increased metastatic potential and poor prognosis. MMPs, especially MMP-2 and MMP-9, are often used as biomarkers for detecting early invasion and metastasis. Elevated MMP expression can help predict the likelihood of cancer spread and the potential for recurrence after treatment[25].
6. Erythropoietin (EPO): EPO is a hormone primarily known for its role in red blood cell production, but it is also regulated by the HIF pathway. EPO is upregulated by HIF-1α under hypoxic conditions and promotes erythropoiesis to increase oxygen delivery to tissues. In breast cancer, the overexpression of EPO has been linked to tumor progression and metastasis. EPO is also thought to exert an anti-apoptotic effect on tumor cells, enhancing their survival in the hypoxic tumor microenvironment. Elevated EPO levels in breast cancer patients may serve as an indicator of tumor hypoxia and a potential marker for poor prognosis, although its clinical utility is still being explored[26].
7. Programmed death-ligand 1 (PD-L1): PD-L1 is a protein expressed on the surface of tumor cells that plays a critical role in immune evasion. Under hypoxic conditions, HIF-1α has been shown to induce the expression of PD-L1, which binds to the PD-1 receptor on T cells and inhibits immune responses. In breast cancer, PD-L1 expression is often associated with an immunosuppressive tumor microenvironment, facilitating immune evasion and promoting tumor growth. The overexpression of PD-L1 in breast cancer has been linked to poor prognosis in some subtypes. However, PD-L1 expression also serves as a predictive biomarker for responsiveness to immune checkpoint inhibitors, particularly in TNBC and tumors with elevated HIF-1α activity[27].
8. Lactate dehydrogenase A (LDHA): LDHA is a key enzyme in the glycolytic pathway, converting pyruvate to lactate under anaerobic conditions. HIF-1α directly regulates the expression of LDHA, promoting the Warburg effect and enabling cancer cells to produce energy through glycolysis rather than oxidative phosphorylation. In breast cancer, high levels of LDHA expression are indicative of a metabolic shift toward aerobic glycolysis, which supports rapid tumor growth and survival. Elevated LDHA levels are associated with poor prognosis, increased tumor aggressiveness, and resistance to chemotherapy. LDHA serves as a potential biomarker for assessing the metabolic status of breast cancer and identifying patients who may benefit from treatments targeting tumor metabolism[28].

**Table 1:** Hypoxia-related biomarkers in breast cancer

Biomarker	Type	Regulated by HIF?	Clinical relevance	Supporting evidence
HIF-1α	Transcription factor	Yes	Prognostic indicator; associated with poor survival, angiogenesis, metastasis	Overexpression in tumor tissues; IHC and plasma studies
HIF-2α	Transcription factor	Yes	Linked to aggressive subtypes and poor prognosis	Observed in ER-negative and basal-like tumors
VEGF	Angiogenic factor	Yes	Marker of tumor angiogenesis and progression	Correlates with microvessel density
CAIX	pH-regulating enzyme	Yes	Poor prognosis; indicator of hypoxia in tumor tissue	Detected via immunohistochemistry
miR-210	microRNA	Yes	Noninvasive diagnostic and prognostic biomarker	Elevated in hypoxic tumors and patient serum
PD-L1	Immune checkpoint protein	Indirectly (via HIF)	Predicts response to immunotherapy; upregulated under hypoxia	HIF-1α binds to PD-L1 promoter in hypoxic TNBC
Glut-1	Glucose transporter	Yes	Associated with poor prognosis; marker of metabolic reprogramming	Overexpression in hypoxic tumor zones
LOX	Enzyme (collagen cross-linking)	Yes	Involved in metastasis and ECM remodeling	Promotes pre-metastatic niche formation

## Therapeutic opportunities targeting the HIF pathway in breast cancer

**Figure 2.**
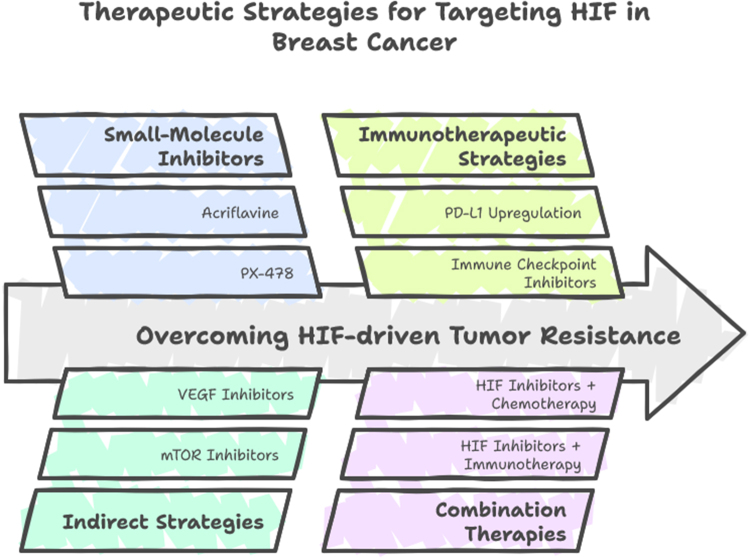
Therapeutic opportunities targeting HIF in breast cancer.

The HIF pathway is a central regulator of cellular adaptation to hypoxia and plays a crucial role in tumor progression, metastasis, angiogenesis, and therapy resistance in breast cancer. As such, targeting the HIF pathway presents a promising therapeutic strategy. Given the involvement of HIF in numerous key aspects of cancer biology, including metabolic reprogramming, immune evasion, and ECM remodeling, several therapeutic approaches have been explored to inhibit HIF activity and its downstream effects. These strategies aim to reduce tumor growth, inhibit metastasis, enhance sensitivity to traditional therapies, and improve patient outcomes (Fig. 2)[2].

1. Inhibition of HIF-1α and HIF-2α stabilization: The first step in the activation of the HIF pathway is the stabilization of the HIF-α subunits (HIF-1α and HIF-2α), which occurs when oxygen levels are low, and prolyl hydroxylases are inactivated. Targeting this stabilization process is one of the most straightforward strategies to inhibit the HIF pathway. Several small molecules have been identified that can inhibit the prolyl hydroxylase enzymes (PHDs), thus preventing the degradation of HIF-α subunits under hypoxic conditions. By restoring the activity of PHDs, these inhibitors prevent the accumulation of HIF-α proteins, thereby blocking the activation of downstream target genes involved in angiogenesis, metabolism, and survival. Drugs such as FG-4592 and Roxadustat, which are PHD inhibitors, are currently being investigated in clinical trials for various cancers, including breast cancer. The use of these inhibitors may help reduce tumor progression and metastasis by disrupting the adaptive responses of cancer cells to hypoxia[21].
2. HIF-1α and HIF-2α antagonists: Another therapeutic approach is the direct inhibition of HIF-1α and HIF-2α activity using specific antagonists. These antagonists work by preventing the dimerization of HIF-α and HIF-β subunits, a necessary step for translocation to the nucleus and subsequent transcriptional activation of target genes. Studies have identified compounds that specifically target the DNA-binding domain of HIF-1α and HIF-2α, blocking their ability to bind to HREs in the promoter regions of target genes. For instance, compounds like YC-1, which is known to inhibit HIF-1α activity, have shown promise in preclinical studies. Similarly, the development of small molecule inhibitors such as PT2385, which targets HIF-2α, is being explored in clinical trials, particularly for tumors like clear cell renal carcinoma, which heavily rely on HIF-2α for their growth. The application of such inhibitors in breast cancer could potentially reduce tumor cell survival and metabolic adaptations in hypoxic regions[29].
3. Targeting HIF-1α downstream effectors: Since HIF signaling regulates a variety of downstream targets involved in key processes such as angiogenesis (VEGF), metabolism (GLUT-1, LDHA), and cell survival (BCL-2), targeting these downstream effectors represents another promising strategy for inhibiting the HIF pathway in breast cancer. Several therapies are being developed to specifically target the products of HIF-1α-induced transcription, such as VEGF inhibitors, which aim to block tumor-induced angiogenesis. Bevacizumab, a monoclonal antibody against VEGF, has shown efficacy in combination with chemotherapy in various cancers, including breast cancer. Targeting other HIF-1α-regulated genes, such as GLUT-1, which promotes the Warburg effect, is also under investigation. Inhibitors of GLUT-1, such as WZB117, have demonstrated potential in preclinical models by decreasing glucose uptake and disrupting the metabolic advantage of tumors. By targeting these downstream effectors, therapies can mitigate the tumor’s ability to adapt to hypoxic conditions and inhibit both tumor growth and metastasis[7].
4. Inhibition of angiogenesis: Anti-VEGF therapies: As mentioned, VEGF is one of the most well-characterized HIF-1α-induced genes involved in angiogenesis. Targeting VEGF signaling is a crucial strategy for disrupting the tumor vasculature and depriving the tumor of nutrients and oxygen. Anti-VEGF therapies, such as bevacizumab (Avastin), have been tested in clinical trials for breast cancer, although their effectiveness as a monotherapy has been limited. However, when combined with chemotherapy, anti-VEGF therapies have shown improved outcomes in certain breast cancer subtypes. Other approaches are focusing on the development of small molecule inhibitors or monoclonal antibodies targeting VEGF receptors, which could further reduce tumor angiogenesis and inhibit metastasis. By inhibiting angiogenesis, these therapies aim to reverse one of the key adaptations that breast cancer cells utilize to survive in low-oxygen environments[30].
5. Targeting metabolic reprogramming in tumors: One of the hallmark features of cancer cells is their ability to reprogram metabolism to support rapid growth and survival. Under hypoxic conditions, HIF-1α promotes the shift toward aerobic glycolysis (the Warburg effect), allowing tumor cells to produce ATP through glycolysis even when oxygen is scarce. Targeting the enzymes involved in glycolysis, such as LDHA, is a promising therapeutic approach. LDHA inhibitors have shown preclinical promise in disrupting the metabolic adaptation of cancer cells and sensitizing them to treatment. Additionally, inhibition of other HIF-regulated metabolic enzymes involved in glucose uptake and lactate production may further deprive tumor cells of the metabolic advantages they require for survival in hypoxic environments. These metabolic therapies could be particularly effective in breast cancer subtypes that exhibit aggressive growth and high glycolytic activity, such as TNBC[31].
6. Immune modulation by targeting HIF: The hypoxic tumor microenvironment also promotes immune evasion by modulating immune cell function. HIF-1α has been implicated in the upregulation of immune checkpoint molecules such as PD-L1, which inhibit anti-tumor immune responses by binding to PD-1 receptors on T cells. Blocking this interaction is the basis for immune checkpoint inhibitors, which have revolutionized cancer treatment, particularly in cancers such as melanoma and lung cancer. Combining HIF inhibitors with immune checkpoint inhibitors could enhance the immune response against breast cancer cells. Targeting HIF-induced immune suppressive pathways may improve the efficacy of immunotherapies and reduce the immune evasion mechanisms that many tumors, including breast cancer, utilize. Combining HIF inhibition with immune checkpoint blockade is a promising area of research that could lead to synergistic therapies for breast cancer[32].
7. Targeting the HIF pathway in tumor stromal cells: In addition to tumor cells, the stromal cells in the tumor microenvironment, including cancer-associated fibroblasts (CAFs) and immune cells, also contribute to HIF signaling. These cells respond to the hypoxic tumor microenvironment by secreting growth factors and cytokines that promote tumor progression. Targeting HIF signaling in these stromal cells may provide an additional therapeutic opportunity. In preclinical models, inhibiting HIF signaling in CAFs has been shown to reduce fibrosis, suppress tumor growth, and improve the efficacy of chemotherapy. Additionally, targeting HIF-regulated cytokines such as interleukin-8, which promotes tumor angiogenesis and immune cell recruitment, could disrupt the supportive tumor stroma and improve therapeutic outcomes[33].
8. Combining HIF inhibition with chemotherapy and radiation therapy: Finally, another promising strategy involves combining HIF inhibitors with traditional therapies such as chemotherapy and radiation. Tumor cells in hypoxic regions are often more resistant to chemotherapy and radiation due to their altered metabolism and enhanced survival pathways. HIF inhibitors can sensitize tumors to these therapies by disrupting the adaptive responses that allow cancer cells to survive under stress. In preclinical studies, the combination of HIF inhibitors with chemotherapeutic agents such as cisplatin and paclitaxel has shown enhanced anti-tumor effects. Similarly, HIF inhibitors may enhance the efficacy of radiation therapy by increasing the sensitivity of hypoxic tumor cells to radiation-induced DNA damage[34].

## Challenges in targeting the HIF pathway in breast cancer

Despite the promising potential of HIFs as diagnostic and prognostic biomarkers in breast cancer, several challenges hinder their widespread clinical application. One major challenge is the heterogeneity of HIF expression across different tumor types and stages, complicating the identification of universally applicable biomarkers. The dynamic nature of the tumor microenvironment and fluctuations in oxygen levels can result in variable HIF activity, making it difficult to establish standardized diagnostic thresholds or reliable biomarkers that work across all patient populations^[35–37]^. Another key hurdle is the lack of reproducibility in detecting HIF-related markers in clinical settings. Although several HIF-regulated proteins and microRNAs have shown promise in preclinical studies, their utility in liquid biopsies or noninvasive methods remains to be fully validated. Further research is needed to ensure the sensitivity and specificity of these markers, as well as their ability to reflect true tumor hypoxia over other physiological changes in the body^[6,38]^.

From a therapeutic standpoint, while the targeting of HIFs holds great potential, there is still a limited understanding of the long-term effects of HIF inhibition. HIFs play crucial roles not only in tumor progression but also in normal physiological processes such as tissue repair and metabolism. Therefore, therapeutic strategies must carefully balance the clinical benefits of HIF inhibition with the risk of off-target effects or adverse reactions, particularly in patients with other comorbidities^[39,40]^. Looking forward, multi-omics approaches integrating genomics, proteomics, and transcriptomics could provide a more holistic view of HIF biology, allowing for the identification of more robust, context-dependent biomarkers. Advances in liquid biopsy technologies and the development of targeted delivery systems could also overcome existing challenges, enabling earlier detection and real-time monitoring of breast cancer^[37,41]^.

## HIFs as emerging diagnostic biomarkers in breast cancer

HIFs, particularly HIF-1α and HIF-2α, are transcriptional regulators that respond to low oxygen levels in the tumor microenvironment. In breast cancer, hypoxia is a common and early event in tumor evolution, and the activation of HIF drives a wide array of changes that promote tumor survival, progression, and resistance to treatment[42]. Recent studies have highlighted the diagnostic potential of HIFs and their downstream effectors. Elevated levels of HIF-1α have been detected in both tissue and plasma samples of breast cancer patients, correlating with tumor size, grade, and lymph node involvement. Additionally, HIF-regulated molecules such as VEGF, CAIX, and GLUT1 are increasingly being investigated as circulating biomarkers. These factors can reflect ongoing tumor hypoxia and may aid in early detection, especially when combined with imaging or other molecular assays^[43,44]^. Moreover, the noninvasive detection of HIF-regulated microRNAs and long non-coding RNAs (e.g. HIF1A-AS2) through liquid biopsy platforms is opening new frontiers in early diagnosis and monitoring of disease progression. Such approaches offer promise in complementing existing diagnostic tools and enabling more personalized cancer care[45]. While still under active research, the use of HIFs as diagnostic biomarkers holds great potential for improving the early detection and clinical management of breast cancer, especially in aggressive subtypes where early intervention is critical (Table 2)[46].

**Table 2:** Clinical trials of HIF in breast cancer

Study type	HIF target/marker	Agent/biomarker	Model/population	Key findings	Clinical relevance
Preclinical	HIF-1α	PX-478	Mouse xenograft model	Suppressed HIF-1α expression; reduced tumor growth and angiogenesis	Promising HIF-1α inhibitor for potential clinical translation
Preclinical	HIF-1α and VEGF	YC-1	Human breast cancer cells	Inhibited HIF-1α transcriptional activity and VEGF expression	Potential dual-targeted antiangiogenic therapy
Preclinical	HIF-1α-regulated genes	miR-210	Cell lines (MCF-7, MDA-MB-231)	Elevated in hypoxic conditions; regulates cell proliferation	Candidate hypoxia-associated circulating biomarker
Clinical (retrospective)	HIF-1α	IHC in tumor tissues	150 breast cancer patients	High HIF-1α levels correlated with poor prognosis and lymph node metastasis	Prognostic biomarker
Clinical (prospective)	HIF-1α	Plasma HIF-1α	80 breast cancer patients vs controls	Elevated levels in patients; associated with tumor burden	Potential noninvasive diagnostic marker
Preclinical	HIF pathway	Acriflavine	Animal model and breast cancer cells	Inhibited HIF-1 dimerization; suppressed tumor growth	HIF-1α/HIF-2α dual inhibitor under investigation
Clinical (phase I/II)	HIF-2α	PT2385, PT2977	Renal cell carcinoma (not yet breast cancer)	Demonstrated target specificity and tolerability	Encouraging results for cross-application in HIF-driven breast cancer
Preclinical	HIF-1α and immune regulation	PD-L1 (HIF-regulated)	TNBC cell lines under hypoxia	Hypoxia-induced HIF-1α upregulated PD-L1 expression; immune evasion enhanced	Implications for immunotherapy response prediction

## Novelty of the review in context of existing literature

While several recent reviews, including the comprehensive work by Luo *et al*[7], have addressed the role of HIFs in breast cancer, the present review adds unique value by specifically focusing on the dual role of HIFs as both diagnostic and prognostic biomarkers, a perspective that has often been overshadowed by the emphasis on therapeutic targeting. Luo *et al*[7] largely emphasized the molecular mechanisms and therapeutic interventions targeting HIF pathways, particularly HIF inhibitors and small molecules in preclinical and clinical stages. However, our review shifts attention to the emerging potential of HIFs in noninvasive diagnostics, highlighting circulating HIF-regulated biomarkers, such as plasma HIF-1α levels, CAIX, and hypoxia-related miRNAs, which are underexplored in existing literature^[45,46]^. Additionally, this review contextualizes HIF-associated immune modulation, such as the influence of HIFs on PD-L1 expression, not only as a prognostic factor but also in terms of diagnostic relevance and treatment responsiveness, which many earlier reviews have not directly correlated. By integrating recent evidence from clinical and translational studies, and mapping out biomarker candidates with practical diagnostic implications, the review offers a clinically aligned perspective that can inform both early detection strategies and risk stratification[47]. This narrative-driven synthesis, supported by graphical models and categorized tables, enhances the translational relevance of the HIF pathway in breast cancer – providing a bridge between bench research and bedside application that complements but clearly diverges in focus and scope from existing reviews like Luo *et al*[7].

## Future directions in targeting the HIF pathway in breast cancer

As our understanding of the HIF pathway and its role in breast cancer progression deepens, several exciting future directions are emerging for the development of more effective therapies. To fully harness the potential of targeting the HIF pathway, the following research and clinical approaches should be pursued:

1. Development of isoform-specific HIF inhibitors: One of the promising future directions in targeting the HIF pathway is the development of isoform-specific inhibitors. HIF-1α and HIF-2α play distinct roles in breast cancer biology, and therapies targeting both isoforms may have different effects depending on the tumor subtype. Recent research has revealed that HIF-2α, in particular, is involved in the progression of certain aggressive breast cancers, including TNBC. Isoform-specific inhibitors may allow for a more targeted approach to treatment, minimizing the potential for off-target effects and improving therapeutic efficacy. Further studies are needed to identify small molecules or biologics that can selectively inhibit one isoform without affecting the other[47].
2. Targeting the tumor microenvironment: Future therapeutic strategies will likely focus on targeting not just tumor cells but also the stromal components of the tumor microenvironment (TME), including the endothelial cells, fibroblasts, and immune cells that interact with hypoxic tumor regions. Since the TME plays a crucial role in the activation and maintenance of the HIF pathway, disrupting the pro-tumorigenic environment may enhance the effectiveness of HIF-targeted therapies. Strategies could include inhibiting the release of growth factors, cytokines, and ECM components that sustain tumor hypoxia and metastasis. Additionally, targeting immune cells in the TME that are modulated by HIF signaling may also promote anti-tumor immunity and improve treatment outcomes^[48,49]^.
3. Combining HIF Inhibition with immunotherapy: Immunotherapy has shown promise in treating various cancers, including breast cancer. The combination of HIF inhibitors with immune checkpoint inhibitors, such as PD-1/PD-L1 blockers, could enhance immune responses and overcome the immune-suppressive effects of tumor hypoxia. HIF signaling is known to contribute to the recruitment of immunosuppressive cells, such as regulatory T cells (Tregs) and myeloid-derived suppressor cells, within the tumor. By targeting HIF signaling in both tumor cells and immune cells, it may be possible to reprogram the immune microenvironment, improve immune surveillance, and enhance the efficacy of immune checkpoint inhibitors. Clinical trials are needed to explore the synergy between HIF inhibitors and immunotherapies in breast cancer treatment^[50,51]^.
4. Nanomedicine for targeted drug delivery: Nanotechnology holds promise in improving the delivery of HIF-targeted therapies to tumor cells, especially in the hypoxic regions where drug penetration is limited. Nanoparticles, liposomes, and other nanomedicines can be engineered to specifically target tumor tissues, allowing for the precise delivery of HIF inhibitors to hypoxic regions. Moreover, nanomedicines can also carry other therapeutic agents, such as chemotherapeutic drugs or siRNA, to further enhance the anti-cancer effects of HIF inhibition. The development of “smart” nanoparticles that release their payload in response to specific tumor microenvironmental factors, such as low pH or hypoxia, may improve the specificity and efficacy of HIF-targeted therapies[52].
5. Personalized medicine and biomarker development: A critical future direction for HIF-targeted therapies in breast cancer is the identification and validation of reliable biomarkers to select patients who are most likely to benefit from treatment. Understanding the tumor’s hypoxic status, HIF pathway activation, and the presence of resistance mechanisms will enable personalized treatment strategies. Liquid biopsy techniques, such as circulating tumor DNA and exosome profiling, could help in monitoring the dynamic changes in tumor hypoxia and HIF activation over time. Additionally, advanced imaging techniques, such as positron emission tomography with hypoxia-specific tracers, may allow for the noninvasive assessment of tumor hypoxia, helping to guide treatment decisions and monitor therapeutic response[53].
6. Targeting HIF-mediated metabolic reprogramming: Cancer cells are known to undergo metabolic reprogramming in response to hypoxia, with HIF playing a central role in regulating glucose metabolism and oxidative stress. Targeting the metabolic pathways regulated by HIF, such as glycolysis, mitochondrial function, and autophagy, could provide an additional therapeutic approach in combination with HIF inhibition. In particular, targeting key enzymes involved in HIF-driven glycolysis, such as lactate dehydrogenase (LDH) and pyruvate kinase M2, may inhibit the metabolic adaptation of cancer cells and enhance the effects of HIF-targeted therapies. Further exploration of the metabolic dependencies of hypoxic tumor cells is needed to identify novel metabolic inhibitors that can complement HIF pathway modulation[54].
7. Clinical trials and combination therapies: As research progresses, the next logical step is to move forward with clinical trials to evaluate the safety, efficacy, and optimal dosing of HIF inhibitors in breast cancer patients. Early-phase clinical trials should assess the combination of HIF inhibitors with standard chemotherapy, targeted therapies, or other novel agents. Clinical studies focused on specific breast cancer subtypes, such as TNBC or HER2-positive tumors, will be crucial for understanding how the HIF pathway contributes to tumor biology in these distinct patient populations. Combination strategies that target multiple aspects of the tumor, including HIF, angiogenesis, and immune modulation, may provide synergistic effects and overcome resistance mechanisms that arise with monotherapy[55].
8. Exploring the role of HIF in cancer stem cells (CSCs): CSCs are thought to play a crucial role in tumor initiation, metastasis, and relapse. The HIF pathway is implicated in maintaining the stemness of cancer cells under hypoxic conditions, which could contribute to therapy resistance. Future research should focus on the role of HIF in breast cancer stem cells (BCSCs) and their potential as a therapeutic target. Targeting HIF signaling in BCSCs could inhibit tumor initiation and metastatic spread, while also improving the sensitivity of CSCs to conventional therapies. Studies on the interaction between HIF and stem cell markers, such as CD44 and ALDH1, may provide valuable insights into CSC-targeted therapies in breast cancer[51].
9. HIF and metastasis: Hypoxia-induced HIF activation is known to drive the metastatic potential of breast cancer cells. HIF promotes the expression of genes involved in EMT, invasion, and metastasis. Targeting HIF-mediated metastasis could have a significant impact on preventing tumor spread to distant organs. Future research should focus on understanding the molecular mechanisms by which HIF induces metastasis and developing strategies to block these processes. Additionally, therapies targeting the vascular remodeling and ECM changes induced by HIF could help limit metastasis by preventing tumor cell invasion and dissemination^[52,53]^.
10. Exploring the use of HIF inhibitors in adjuvant therapy: HIF inhibitors may hold promise as adjuvant therapies for patients undergoing surgery, radiation, or chemotherapy. By targeting the residual hypoxic tumor cells that survive initial treatment, HIF inhibitors could reduce the likelihood of tumor recurrence and metastasis. Incorporating HIF inhibitors into adjuvant therapy regimens could also help normalize the tumor vasculature, enhancing the delivery of other therapies and improving their effectiveness. Clinical trials investigating the use of HIF inhibitors in the adjuvant setting, in combination with standard treatments, will be essential for determining the long-term benefits and potential risks of these therapies^[54,55]^.

## Conclusion

The HIF pathway plays a pivotal role in the progression of breast cancer by enabling tumor cells to adapt to the challenges of hypoxia, which is common in rapidly growing tumors. Through its regulation of various biological processes such as angiogenesis, metabolism, invasion, and immune modulation, HIF contributes to tumor growth, metastasis, and resistance to conventional therapies. As a result, targeting the HIF pathway presents a promising therapeutic strategy for improving breast cancer treatment outcomes. Recent advances in understanding the molecular mechanisms of HIF activation have opened new doors for targeted therapies. These therapies, which include HIF inhibitors, isoform-specific molecules, and strategies targeting the TME, are currently being explored in preclinical and clinical settings. Biomarkers associated with HIF signaling, such as VEGF and GLUT1, may help identify patients most likely to benefit from these therapies, enabling more personalized treatment approaches. Additionally, combining HIF-targeted therapies with immunotherapy, chemotherapy, or other emerging modalities may enhance their therapeutic effects and overcome potential resistance mechanisms.

## Data Availability

Not applicable as this is a narrative review.

## Research registration unique identifying number (UIN)

Not applicable as this is a narrative review.
